# Snowball Sampling Study Design for Serosurveys Early in Disease Outbreaks

**DOI:** 10.1093/aje/kwab098

**Published:** 2021-04-08

**Authors:** Lee Kennedy-Shaffer, Xueting Qiu, William P Hanage

**Affiliations:** 1 Department of Mathematics and Statistics, Vassar College, Poughkeepsie, New York, United States; 2 Center for Communicable Disease Dynamics, Harvard T.H. Chan School of Public Health, Boston, Massachusetts; 3 Department of Epidemiology, Harvard T.H. Chan School of Public Health, Boston, Massachusetts

**Keywords:** asymptomatic infection, contact tracing, coronavirus disease 2019, design effect, SARS-CoV-2, serosurvey sampling, transmission chain

## Abstract

Serological surveys can provide evidence of cases that were not previously detected, depict the spectrum of disease severity, and estimate the proportion of asymptomatic infections. To capture these parameters, survey sample sizes may need to be very large, especially when the overall infection rate is still low. Therefore, we propose the use of “snowball sampling” to enrich serological surveys by testing contacts of infected individuals identified in the early stages of an outbreak. For future emerging pandemics, this observational study sampling design can answer many key questions, such as estimating the asymptomatic proportion of all infected cases, the probability of a given clinical presentation for a seropositive individual, or the association between characteristics of either the host or the infection and seropositivity among contacts of index individuals. We provide examples, in the context of the coronavirus disease 2019 (COVID-19) pandemic, of studies and analysis methods using a snowball sample and perform a simulation study that demonstrates scenarios where snowball sampling can answer these questions more efficiently than other sampling schemes. We hope such study designs can be applied to provide valuable information to slow the present pandemic as it enters its next stage and in early stages of future pandemics.

ORIGINAL UNEDITED MANUSCRIPT

ABBREVIATIONS

COVID-19: coronavirus disease 2019
ICC: intracluster correlation coefficient
SARS-CoV-2: severe acute respiratory syndrome coronavirus 2

ORIGINAL UNEDITED MANUSCRIPT

There is great interest in the results of serosurveys based on antibodies against the severe acute respiratory syndrome coronavirus 2 (SARS-CoV-2) virus, to indicate the true numbers of people infected so far in the pandemic, the proportion that might be immune in future waves of infection, and the proportion of people infected who experience mild or no symptoms. These and many other parameters important for the coronavirus disease 2019 (COVID-19) pandemic public health response were difficult to estimate early in the pandemic. In addition, while asymptomatic or pre-symptomatic cases are known to be capable of transmission, the proportion of infections caused by such cases is not clear. While it appears that SARS-CoV-2 transmission displays overdispersion, a high variance in secondary cases per index case around the population mean basic reproduction number ($R_0$), we do not yet know what characteristics are associated with superspreading events (1–3).

In addition to estimating population immunity levels, serosurveys can be used to determine the proportion of infections that might have been undetected because they were either minimally symptomatic or exhibited symptoms that did not lead to testing. While random testing is common for such surveys, the cumulative incidence will vary greatly depending on the stage of the pandemic, and a very large sample may be necessary to obtain enough cases to capture less common disease presentations. An alternative study design based on “snowball sampling” offers a route to collect data on the spectrum of clinical severity and transmissibility. This sampling approach is a form of survey sample enrichment for hard-to-reach populations (4,5). While enriched sampling might typically focus on a marginalized or underrepresented community, snowball sampling in this context enriches for the presence of seropositivity. Similar enrichment can occur in studies focused on high-risk populations (e.g., health care workers (6)) or by studying family members of infected individuals (7), but these are not as representative of the population and possible transmission routes in a more general setting.

Snowball sampling, or “chain referral sampling,” has a rich history, especially in the sociology research literature (8–10). The general research method involves identifying index individuals and, along with collecting information on them, asking them to refer other individuals suitable for the study (8,10). These named individuals are then recruited into the study. This process may end there or continue for further stages (9). The method is often used for qualitative studies of “hidden” populations, where subjects are difficult to reach or a sufficient sample is unlikely to be obtained from random sampling (5,10,11). It has more recently been used in infectious disease settings as a cost-effective way to recruit individuals into care and into studies (12–14). One study of human immunodeficiency virus infection found it to be a more cost-effective approach and generate a more representative sample of the spectrum of disease than standard recruitment methods (14). The generalizability of results from snowball sampling has been criticized because the sample is not a true probability sample (11,15,16). This can be mitigated, however, by ensuring a random or representative selection of index individuals and by accounting for clustering in the analysis (15–18).

ORIGINAL UNEDITED MANUSCRIPT

By taking people who are known to have been infected and tracing their contacts in order to identify possible transmission events, we are able to both estimate how many secondary cases were infected by an index case and obtain a larger dataset of individuals who have been infected, as well as individuals who were contacts of the same index individual but not infected. The goal of snowball sampling, as we propose it, is not to determine the amount of population-level immunity, which is best addressed by conventional serosurveys, but to obtain many individuals who have been exposed in order to estimate the range of clinical presentations and their relation to transmission. We propose that investigators consider a snowball sampling approach based on contact tracing in order to more fully answer important questions about the clinical presentations of disease and factors associated with transmission in a cost-effective way.

## PROPOSED APPLICATION OF SNOWBALL SAMPLING FOR SARS-CoV-2 SEROLOGICAL SURVEYS

We propose to apply the snowball sampling methodology to enrich survey samples in outbreaks for seropositive individuals. This relies on contact tracing, which is primarily used for outbreak mitigation and to identify linkages between transmission chains (19). Assuming that a serological assay with high specificity and sensitivity for previous SARS-CoV-2 infection is available, the method begins with a sample of individuals who were infected. We then proceed to test the reported contacts of these cases, both those to whom the primary case is known to have transmitted and other potential contacts who may not have been previously identified. Backward tracing, which incorporates potential infectors of the index case, may also be used. If feasible, virologic testing can also be used to identify contacts with active infection who may not yet be

ORIGINAL UNEDITED MANUSCRIPT

seropositive. The sample can be enlarged either by adding more index cases and their contacts, or by adding additional layers (contacts of contacts who test positive). Enlargement continues until the sample is estimated to have sufficient statistical power to answer the question(s) of interest. A history of symptoms relevant to the question of interest, covering the time period from the earliest possible infection date to the latest possible date yielding seropositivity, is collected for each identified case and contact.

## POTENTIAL APPLICATIONS AND QUESTIONS OF INTEREST

Several types of scientific questions of interest may be answered through this design. For example, using only sampled individuals who test positive:

1. Identify the range of clinical presentations for an individual with a positive serology test (seropositive individual).
2. Identify the probability of a given course of disease for a seropositive individual.
3. Identify the association between a contact's characteristics of interest (e.g., types of contact or personal characteristics) and their course of disease. For instance, some individuals may be more likely to experience minimally symptomatic infections.

With information on both seropositive and seronegative contacts, researchers can also answer questions about transmission such as:

4. Identify the association between some characteristics of interest (of either the contact or index individual) and seropositivity among contacts of index individuals, to determine which are associated with increased likelihood of transmission. For example, cases who are asymptomatic (and were undetected by initial contact tracing) may be more likely to transmit because they are unaware of their status.

ORIGINAL UNEDITED MANUSCRIPT

Table 1 provides examples of these four types of questions that are relevant for outbreaks of infectious diseases such as COVID-19, along with analysis methods that are appropriate for the snowball sampling design.

Other questions of interest may focus on the network structure itself, that is, the number of seropositive contacts of each index individual. Much of the snowball sampling literature focuses on these types of questions and we refer readers there for appropriate analysis methods (8,9,15).

STATISTICAL ANALYSIS

The primary difference in analysis between random sampling (as in standard serosurveys) and snowball sampling is that, in snowball sampling, estimation and inference must account for the clustered nature of the data. The contacts of an individual have a potentially shared exposure (and perhaps other latent shared characteristics) and thus can be viewed as a cluster (20,21). We will assume for now that index individuals are a random sample of all possible index individuals from some larger population of interest (e.g., all individuals with confirmed infection in a given time range at a given geographic location, workplace, etc.). This could occur if the index individuals are identified through random surveys of the population or routine surveillance with a very sensitive test. This allows inference to proceed, treating the sample as a cluster sample from a larger population of clusters (22).

For questions 1 and 2, analysis can account for this potential correlation by using a hierarchical model, where the probability of a given clinical presentation differs depending on the index

ORIGINAL UNEDITED MANUSCRIPT

individual. A common assumption is that the cluster-specific probabilities are independent and identically distributed according to some distribution with mean $\pi$ and variance $\sigma^2$ (23,24). This is often parameterized using the intracluster correlation coefficient (ICC), $\varrho = \frac{\sigma^2}{\pi(1-\pi)}$. Details of this approach are given in the Web Appendix.

For question 3, we can use logistic or multinomial logistic regression approaches that account for the clustering of the data (22). Two common approaches are mixed effects models and fitting regression models using generalized estimating equations (20,21,25). Both approaches allow for the specification of either individual-level (i.e., characteristics of the contact or the test used) or cluster-level (i.e., characteristics of the index case) covariates of interest in the model. Generalized estimating equations have the advantage of being robust to misspecification of the correlation model (21), for example if some of the individuals identified by an index individual were actually infected by someone else. For mixed effects modeling approaches, clustering parameters may not be interpretable if contacts include directly infected individuals, individuals infected by another source, and potential infectors of the index individual. However, this is equivalent to misspecifying the random effects distribution, which in general has minimal effect on estimation and inference (26). Additional details can be found in the Web Appendix.

Finally, for question 4, we are using not only the characteristics of the seropositive contacts but also those of the seronegative contacts. This mimics a case-control study design matched by index individual and thus can be analyzed similarly to other matched case-control studies; methods include Cochran-Mantel-Haenszel analysis stratified by index individual and conditional logistic regression models (25,27–30). Using a snowball sample as the basis for a

ORIGINAL UNEDITED MANUSCRIPT

matched case-control study was shown to perform well and avoid selection bias in a study on drug abuse (18).

The above methods only use data from the contacts. For questions 1, 2, and 3, however, they can be easily extended to include the index individuals themselves and second-order contacts; i.e., contacts of contacts of index individuals. In these cases, multiple random effects terms or more complex correlation structures may need to be specified. Depending on the question of interest, using more waves in snowball sampling can ensure greater sample diversity (17) but may introduce more difficulty for valid statistical inference (15).

We have so far assumed that the index individuals are a random sample of individuals infected with SARS-CoV-2. If, however, the index individuals over- or under-represent certain clinical presentations (e.g., index individuals are selected among individuals with symptomatic COVID-19) and the clinical presentation of an index case is related to the clinical presentation of their infected contact, the methods presented here will not represent the full population (5,16). To account for this, a systematic sample of index individuals can be used that represents all clinical presentations or factors associated with them, ensuring diversity of index cases (17). We can adjust the methods appropriately by using stratified analyses based on the factors used in sampling the index individuals (21,22,31).

SAMPLE SIZE AND POWER CALCULATIONS

Making the assumptions described in the Statistical Analysis section, we can calculate the required sample size according to the analysis method for various questions.

ORIGINAL UNEDITED MANUSCRIPT

For question 1, we can calculate the required sample size (given an estimate of the number of seropositive contacts per index individual) by finding the number of index individuals that gives a specific probability of observing at least one contact with the clinical presentation, assuming a true underlying probability for that presentation. Clustering can be accounted for by specifying the ICC $\rho$. For questions 2 and 3, we must inflate the variance (and thus the required sample size) of a standard analysis by an appropriate design effect. This design effect can be estimated by $DE = 1 + (\bar{m} - 1)\rho$, where $\bar{m}$ is the average number of seropositive contacts per index individual (21,22,32). More details can be found in the Web Appendix.

Note that for questions 1–3, the outcome of interest is not seropositivity, but rather the clinical presentation itself. This likely has a lower ICC than seropositivity. In addition, some infected individuals will have been infected by someone other than the index individual, so the hierarchical model may not be correctly specified. Sample size calculations are thus likely to be conservative, as the true correlation will be lower than the hypothesized correlation. For analyses with an individual-level covariate, precise methods for sample size estimation are not available, but the design effect using an appropriately-adjusted ICC may be a reasonable approximation (21,24,31). More complex sample size formulae can be used if the analysis has multiple levels of clustering or stratification (21,22,31).

We can use these formulae to compare the required sample size for a snowball sample to that for a simple random sample. The inflated variance of the design is counterbalanced by the higher percentage of tested individuals who are seropositive due to the enriched sample from this

ORIGINAL UNEDITED MANUSCRIPT

design. The effective sample size of a snowball sample is the number of identified seropositives divided by the design effect. This can be compared to the number of identified seropositives from a random serosurvey to determine the relative efficiency of the two designs. An example of this calculation is given in the Web Appendix.

For question 4, sample size and power calculation methods are available for matched case-control studies (33,34). No simple comparison exists for the relative efficiency of these matched approaches and approaches based on a random sample.

EXAMPLE: SIMULATED STUDY

To illustrate the use of this study design, consider a study that aims to identify a certain symptom of the disease and estimate the percentage of infected individuals who experience that symptom (akin to questions 1 and 2 above). One design would be a random-sample serosurvey where investigators ask individuals who test positive whether they have experienced this symptom. The seropositive individuals form the sample, and the proportion of these who experienced that symptom can be used as an estimate of the symptom rate. Inference can proceed using standard methods for binomial proportions.

Using snowball sampling, instead, a small number of index individuals who were known to be infected are asked to identify contacts during their potential infectious period. These contacts are tested and asked whether they have experienced the symptom. The seropositive contacts form the sample. The proportion of these who experienced that symptom, corrected to account for

ORIGINAL UNEDITED MANUSCRIPT

clustering, can be used as an estimate of the symptom rate. Inference can proceed using clustered survey sample methods for binomial proportions (22,35).

To illustrate the potential benefits of snowball sampling, we present results from a simulation study using a susceptible-exposed-infectious-recovered model in a population of 10,000 individuals. Each individual in the population has a set of daily contacts, with an average of 20. A basic reproduction number of 2.5 is used with a dispersion parameter $k = 0.1$. Both the number of contacts an individual has and their "infectiousness" contribute to the overdispersion of transmission. For simplicity, infected individuals are assumed to be infectious for 8 days beginning with the second day after infection, with an equal probability of infecting a contact on each of those days. On average, 5% of individuals experience the symptom of interest, with additional variation due to a risk factor. Parameters used are summarized in Web Table 1.

We conduct 250 simulations per parameter combination, varying both the sampling time and the ICC of symptoms among infector and infectee. Note that by "sampling time" here we are referring to the last time at which an individual could become infected and test positive on the serological assay. So the actual time at which testing occurred may be weeks later; incorporating virologic testing as well would reduce the need for this wait time.

In each simulated outbreak, at the designated sampling time, we sample and test 600 individuals at random for the "Regular Sampling" approach; and 30 index individuals, who were infected at least 10 days (i.e., the maximum generation interval) prior to the sampling time, for the "Snowball Sampling" approach. All of the contacts of the index individuals are then tested, for

ORIGINAL UNEDITED MANUSCRIPT

an average of 600 tested contacts. We also construct a “Snowball, Error” sample, where each index individual misses two of their true contacts and identifies two false contacts. For each sample, we estimate the symptom rate and construct a 95% confidence interval using a logit transformation approach, appropriately adjusted for clustering by index individual for the snowball samples (35). For simplicity, we conservatively exclude any individual who is identified as a contact by multiple index individuals, although methods for overlapping clusters could be used instead. R code to replicate this simulation study or apply the sampling methodology to a more specific simulation setting is available at https://github.com/leekshaffer/snowball-sampling.

First, to see how well the methods identify this symptom, we can compare the percentages of each simulation with at least one symptomatic individual in the sample. At the earliest sampling time, when the population prevalence is around 5%, only 77% of the regular samples included at least one symptomatic individual, while over 97% of the snowball samples did. Incorporating contact recall error in the snowball samples reduced this percentage by less than 1%.

To compare performance on question 2, estimation of the symptom rate among infected individuals, Figure 1 displays the number of infections identified in each sample (panels A,B), the median and interquartile range of the estimated symptom rate (panels C,D), and the root mean squared error of estimation (panels E,F) across all simulations for each method, by sampling time and ICC. All methods provide unbiased estimation of the true symptom percentage at the sampling time. The snowball samples, with or without contact error, identify more infections, however, and so have a smaller interquartile range and lower root mean squared

ORIGINAL UNEDITED MANUSCRIPT

error than the regular samples. Figure 2 shows the 95% confidence interval width (panels A,B) and empirical coverage (panels C,D) across all simulations for each method by sampling time and ICC. All methods achieve nominal coverage, but the snowball sample estimates have narrower confidence intervals, indicating higher precision. The differences are less pronounced at later sampling times (where the background prevalence is higher) and when the ICC is higher (where the clustered analysis lowers precision). The highest ICC considered, 0.10, is shown in Figure 3. Simulations with less overdispersion of transmission demonstrate similar results (see Web Figures 1–6).

This simulation demonstrates the potential value of snowball sampling in increasing the precision of estimating the symptom rate among infected individuals by enriching the sample for infected individuals. It also demonstrates that minor violations of assumptions, such as some incorrect contact identification, do not negate the benefit of snowball sampling. The approach described here is agnostic to the true infector of a contact, so the method is also robust both to the index individuals including their infector as a contact and to other sources of infection being included among identified contacts for the ICCs studied here.

This simulation study is limited by its simplicity; more complex models for parameters of interest, contact matrices, and transmission parameters can be incorporated to assess the benefits of snowball sampling in a specific setting, for COVID-19 or another disease. It also does not account for imperfect testing sensitivity and specificity and delays in seroconversion, although these would affect both the regular and snowball samples.

ORIGINAL UNEDITED MANUSCRIPT

DISCUSSION

This study design has a number of advantages since its contact-based testing method enriches the sample for cases of infection. This allows us to more rapidly and efficiently determine the range of clinical presentations, including among hard-to-reach individuals who may not have had contact with health care providers (14). In a sample of sufficient size, we would also be able to compare the numbers of onward infections associated with different clinical presentations. More data on the role of asymptomatic and less severe clinical presentations in onward transmission is critical to designing appropriate responses, as existing studies may reflect changing contact patterns due to public awareness of the disease rather than biological patterns of infectiousness (36). This sampling approach could also inform estimates of the secondary attack rate of symptomatic and asymptomatic cases, improving future modeling studies and providing context for tailored public health interventions. As in all studies of secondary attack rates, appropriate definition of contacts is crucial to obtaining unbiased estimates (37).

Compared to other enriched designs, this approach is not limited to certain segments of the population and thus provides a more representative sample of clinical presentations and demographics. It also provides a larger and more representative sample per index individual than only sampling household members, enabling better power to answer a wider range of questions of interest.

There are settings where the snowball sampling design is not feasible, however, or where it has minimal benefits compared to random sampling. First, it is not suited to estimate overall population seroprevalence. Second, it is less beneficial in a more mature epidemic, where the

ORIGINAL UNEDITED MANUSCRIPT

higher overall prevalence reduces the efficiency of the snowball sample enrichment. In a later epidemic, non-pharmaceutical interventions may have also reduced the reproduction number, thus reducing the number of seropositive individuals per index case. These interventions (e.g., social distancing) may also reduce contacts, however, thus preserving the relative benefit of snowball sampling. Third, if there is substantial misclassification of close contacts or bias in recall by index individuals, this can limit the benefits of the design and even lead to bias. In particular, if index individuals are more likely to recall contacts who did experience symptoms, this approach may lead to overestimation of the rates of severe clinical presentations. Finally, a high proportion of superspreading events will lead to high variance in cluster sizes, reducing the effective sample size of the snowball sample.

From the practical side, there is a cost of identifying and reaching index individuals and their close contacts above that of identifying and reaching randomly sampled individuals. This may reduce the number of individuals who can be sampled. This is particularly the case if a true random sample of index individuals is desired so that the results are generalizable, rather than using a convenience sample of index individuals (5). The use of pre-existing contact tracing information from public health authorities will reduce the contact tracing labor required and thus the cost of snowball sampling. It will also reduce contact recall bias by shortening the time delay. Further work should consider whether contact tracing efforts with virologic testing provide enough information to be used as *de facto* snowball samples for retrospective analysis.

As in all serological surveys, the results depend on the sensitivity and specificity of the assay used. Low sensitivity will lead to the exclusion of cases, reducing the sample size achieved and

ORIGINAL UNEDITED MANUSCRIPT

potentially reducing the representativeness of clinical presentations. Low specificity will lead to the inclusion of non-infected individuals as index individuals, resulting in wasted resources, and could also lead to bias in determining the association between characteristics and the likelihood of infection among contacts. By enriching the sample through snowball sampling, resources that might otherwise go to sampling individuals could go to the use of a more accurate test, mitigating some of these problems. If these test characteristics are known, then analysis methods can be adjusted to account for them.

Since directionality is important in identifying transmission risk factors but unable to be fully established in general in such surveys, evaluations of factors that predict transmission should be interpreted with caution (37). In some cases, limiting the analysis to cases where temporal ordering of infection can be established may be warranted.

Finally, the symptom or risk factor being accurately recalled by the close contacts is also important for obtaining unbiased results. This will depend upon the time between their possible infection and the occurrence of the survey, as well as the specificity of the symptom under investigation. For many of these factors, surveys early in the epidemic will perform better than those later in the epidemic, and so snowball sampling might be an effective approach at ensuring adequate power.

The snowball sampling survey design can collect samples in a more rapid and efficient manner than conventional serosurveys, especially in the early stage of an epidemic. Studies using this design can then provide vital information on important parameters, including the range and

ORIGINAL UNEDITED MANUSCRIPT

likelihood of clinical disease severity among infected individuals. It should be considered for use in locations still in the early stage of the COVID-19 pandemic and its properties should be further studied so the method can be improved and used in future infectious disease outbreaks.

DATA AVAILABILITY

All simulated data generated for this study, as well as R code to reproduce the simulations, analyses, and figures, are available at https://github.com/leekshaffer/snowball-sampling.

TABLES

Table 1. Examples of Scientific Questions of Interest

| Question Class | Example Question of Interest | Analysis Methods for Snowball Sampling | Sample Size Considerations | References for Analysis and Sample Size Methods |
|---|---|---|---|---|
| 1. Identify the Range of Clinical Presentations in Seropositive Individuals | Do individuals infected with COVID-19 experience severe joint pain? | Identify any such presentations among identified index individuals and contacts | Clustering of clinical presentations by infector | (20–22) |
| 2. Identify the Probability of a Given Course of Disease in Seropositive Individuals | What proportion of individuals infected with COVID-19 experience anosmia? | One-stage cluster sampling ratio estimation using a hierarchical model | Adjust binomial proportion estimation methods by appropriate design effect based on hypothesized intracluster correlation coefficient | (20–25,35) |
| 3. Identify the Association between Personal Characteristics and Course of Disease among Seropositive Contacts | Is diabetes associated with an increased risk of hospitalization among individuals infected with COVID-19? | Logistic or multinomial logistic regression model, adjusted for clustering by index individual using a hierarchical mixed effects model or fit with generalized estimating equations | Sample size calculations for mixed effects models or generalized estimating equations fits, using a hypothesized intracluster correlation coefficient | (20–26,31,38–40) |
| 4. Identify Association between Characteristics of Index Individual or Contacts and Seropositivity among Contacts | Is age of the index individual associated with an increased risk of transmission to identified contacts? | Cochran-Mantel-Haenszel analysis and conditional logistic regression as used for analysis of matched case-control studies | Matched case-control study sample size methods | (18,25,27–30,33,34) |

COVID-19: Coronavirus disease 2019.

ORIGINAL UNEDITED MANUSCRIPT

FIGURES

Figure 1. **Simulation results for estimated symptom rates.** Median and interquartile range (IQR) of the number of infections per sample (A,B), median and IQR of the estimated symptom rate (C,D), and root mean squared error (RMSE) of the estimated symptom rate (E,F) are compared by sampling time (day 25, 35, and 45), intracluster correlation (ICC) of infector and infectee symptom status (ICC = 0 in A,C,E and ICC = 0.05 in B,D,F), and sampling method, with the default dispersion parameter k = 0.1. The underlying probability of being symptomatic given infection is 5% (vertical line in C,D). All symptom rates are estimated using the logit transformation; estimates for the two snowball samples are adjusted for clustering by the index individual, with contacts named by two or more index individuals removed. Results are from 250 simulations per parameter combination.

Figure 2. **Simulation results for inference on estimated symptom rates.** Median and interquartile range (IQR) of the 95% confidence interval (CI) width (A,B) and empirical coverage (C,D) of the 95% CIs for the symptom rate among infected individuals are compared by sampling time (day 25, 35, and 45), intracluster correlation (ICC) of infector and infectee symptom status (ICC = 0 in A,C and ICC = 0.05 in B,D), and sampling method, with the default dispersion parameter k = 0.1. The nominal CI coverage is 95% (vertical line in C,D). Results are from 250 simulations per parameter combination.

Figure 3. **Simulation results for estimation and inference of symptom rates for intracluster correlation (ICC) of 0.10.** Median and interquartile range (IQR) of the number of infections per sample (A), median and IQR of the estimated symptom rate (B), root mean squared error (RMSE) of the estimated symptom rate (C), median and IQR of the 95% confidence interval (CI) width (D) and empirical coverage (E) of the 95% CIs for the symptom rate are compared by sampling time (day 25, 35, and 45) and sampling method, with the default dispersion parameter k = 0.1 and with ICC = 0.10. The underlying probability of being symptomatic given infection is 5% (vertical line in B) and the nominal CI width is 95% (vertical line in E). All symptom rates are estimated using the logit transformation; estimates for the two snowball samples are adjusted for clustering by the index individual, with contacts named by two or more index individuals removed. Results are from 250 simulations per parameter combination.

ORIGINAL UNEDITED MANUSCRIPT

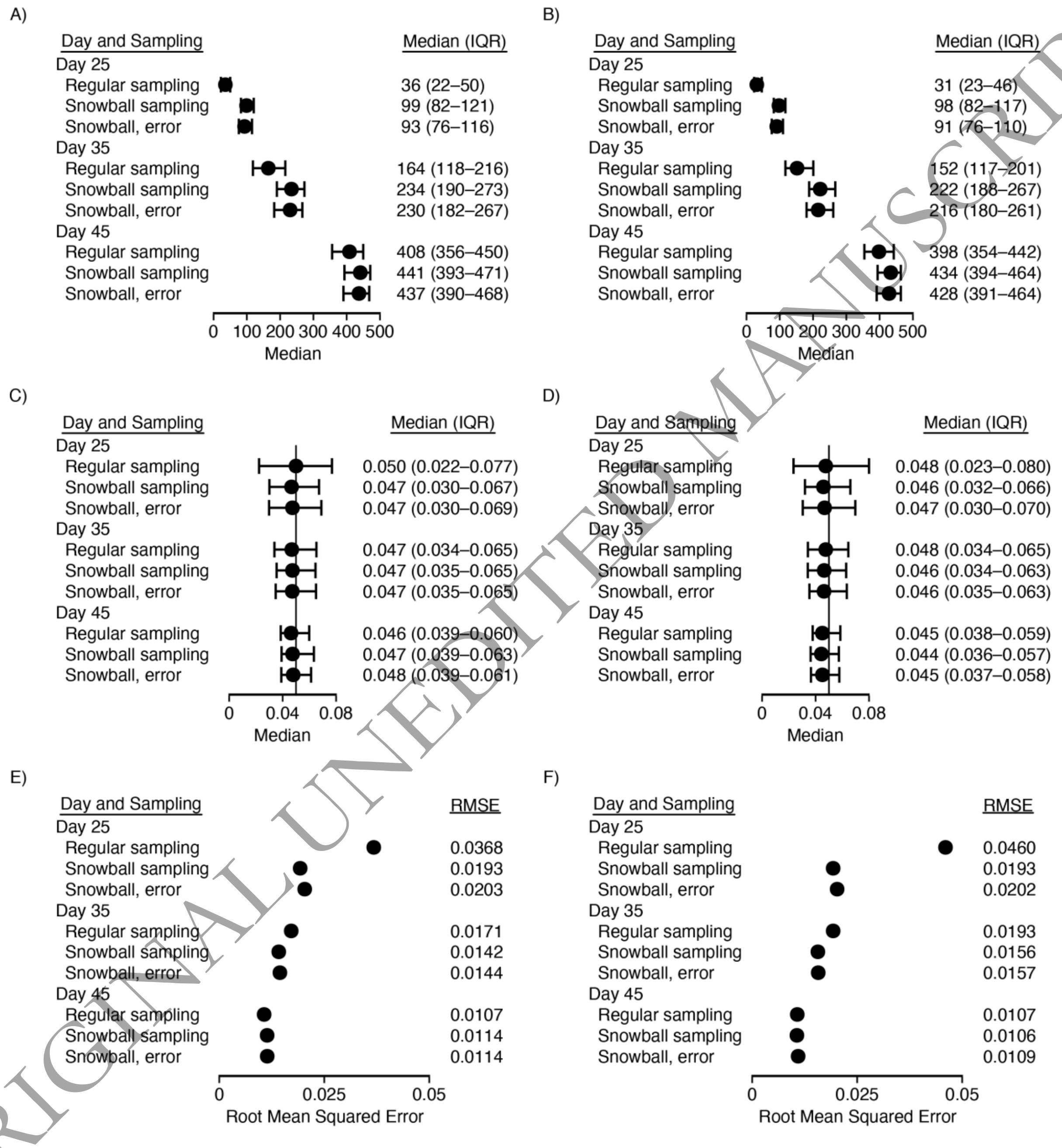

ORIGINAL UNEDITED MANUSCRIPT

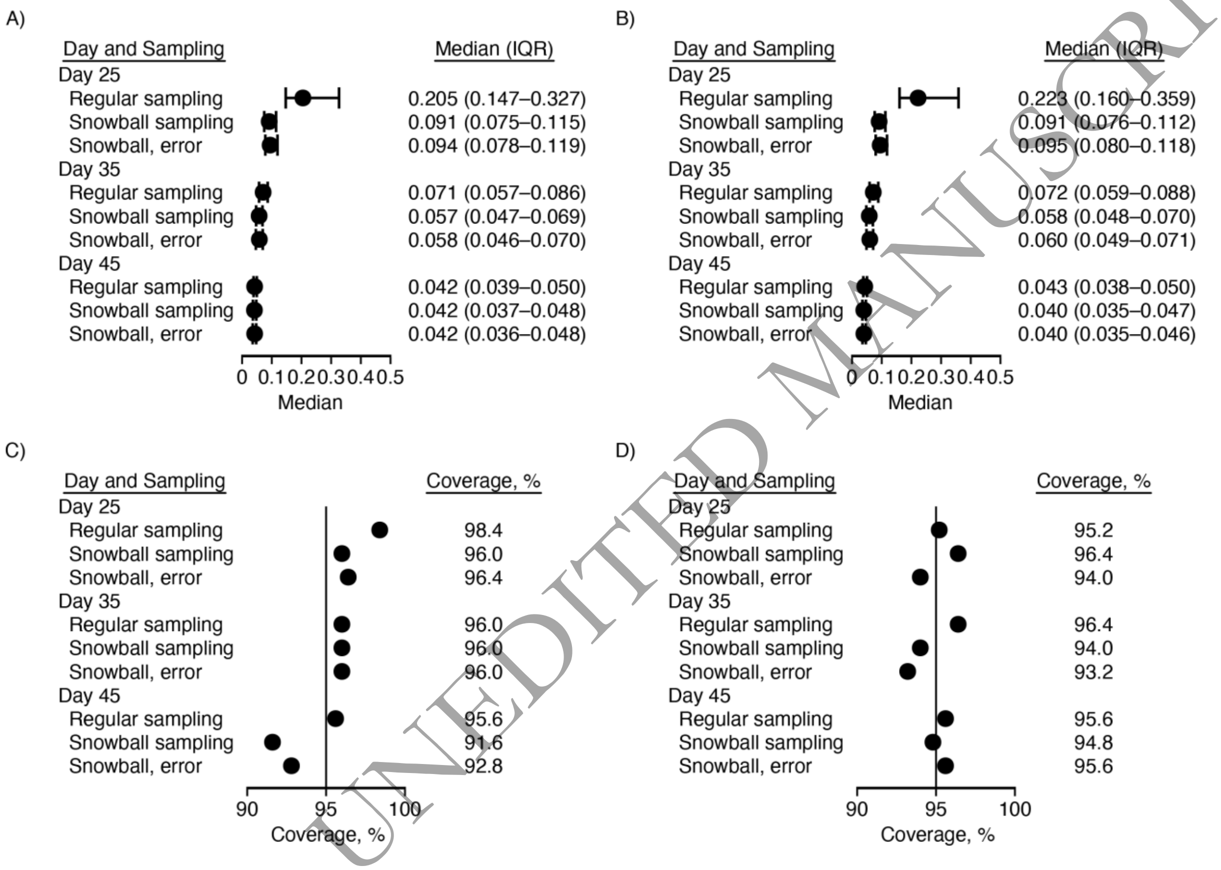

ORIGINAL UNEDITED MANUSCRIPT

ORIGINAL UNEDITED MANUSCRIPT
