# Supplementary material for: Snowball Sampling Study Design for Serosurveys Early in Disease Outbreaks
Source: Am J Epidemiol. 2021 Apr 8;190(9):1918–27. doi: 10.1093/aje/kwab098 (PMC8083564; doi:10.1093/aje/kwab098)
Supplement: Web_Material_kwab098 [file web_material_kwab098.pdf]

WEB MATERIAL

Snowball Sampling Study Design for Serosurveys Early in Disease Outbreaks

Lee Kennedy-Shaffer, Xueting Qiu, and William P. Hanage

Contents:

Web Appendix
Web Table 1
Web Figures 1–6

WEB APPENDIX

Additional Details of Analysis Methods and Sample Size Calculations

Analysis Method Details. In this framework, the key statistical issue for question 1 is the likelihood that a clinical presentation that occurs in a certain underlying proportion of the population will occur in a snowball sample of a given size. Suppose we have n index individuals and, for the ith index individual, we identify m_i seropositive contacts. Let $M = \sum_{i=1}^{n} m_i$ be the total number of seropositive contacts. Assume that within the ith group of contacts (referred to hereafter as "clusters"), the probability of exhibiting a certain clinical presentation is π_i and that, conditional on being in this cluster, each contact's presentation is unrelated to the clinical presentation of any other individual in that cluster. Then the probability that a specific presentation appears in a sample is given by $p_{sampled} = 1 - \prod_{i=1}^{n} (1 - \pi_i)^{m_i}$.

Question 2 concerns ratio estimation from a one-stage cluster sampling survey, and estimation and inference can proceed by accounting for the differing cluster sizes (1,2). Using the same notation as for question 1 and letting Y_{ij} be an indicator of whether the presentation of interest occurs for the jth seropositive contact of the ith index individual, we can estimate the probability of a given clinical presentation from the data by:

$\hat{\pi} = \frac{\sum_{i=1}^{n} \sum_{j=1}^{m_i} Y_{ij}}{\sum_{i=1}^{n} m_i}$, with $Var(\hat{\pi}) = \left(\frac{1}{M}\right)^2 \sum_{i=1}^{n} Var\left(\sum_{j=1}^{m_i} Y_{ij}\right)$.

To make the analysis methods for questions 1 and 2 more tractable, we can make certain assumptions about the distribution of the cluster-specific probabilities π_i. A common assumption is a hierarchical model, assuming that these probabilities are independent and identically

distributed according to some distribution with mean π and variance σ^2(3,4). Under such an assumption, then, to a second-degree approximation in terms of π and σ^2:

$p_{sampled} \approx M\pi - \frac{1}{2}M(\bar{m}-1)(\pi^2 + \sigma^2)$.

For question 2: $Var(\hat{\pi}) = \frac{1}{M}\pi(1-\pi) + \frac{1}{M^2}\sigma^2 \sum_{i=1}^{n} m_i(m_i - 1) \approx \frac{\pi(1-\pi)}{M}[1 + (n-1)\rho]$,

where $\varrho = \frac{\sigma^2}{\pi(1-\pi)}$is the intracluster correlation coefficient (ICC) for a hierarchical model (3,4) and the approximation is exact if $m_i = \bar{m}$, the mean number of seropositive contacts per index individual, for all i (5). Estimation and inference (including hypothesis testing and confidence interval construction) can thus proceed from these results. A full binomial likelihood model can be used to jointly estimate π and σ^2 if, as is common, the nuisance parameter σ^2 is not known.

For question 3, akin to the hierarchical model discussed above, a mixed effects model allows the specification of a random effect for each cluster in addition to the covariates of interest in the model. A parametric assumption about the form of this random effect must be specified (e.g., a normal distribution) and the variance parameter is estimated as a nuisance parameter (6,7). The ICC can be calculated as the ratio of this variance parameter to the marginal variance of the outcome of interest (3,4). For multinomial estimation (i.e., interest in the association between a characteristic and the full set of clinical presentations), mixed-effects multinomial logistic regression can be used (8).

Generalized estimating equations allow for the specification of a logistic regression and a working covariance matrix between individuals within the same cluster (9). This avoids the need to specify a parametric random effect, and the robust variance estimator ensures valid inference regardless of misspecification of the covariance matrix (i.e., the correlation between contacts of the same

index individual need not be known) (9). The ICC is estimated by the procedure as a nuisance parameter.

Sample Size with Varying Cluster Sizes. For questions 2 and 3, when the number of seropositive contacts per index individual varies substantially, better (and more conservative) estimates of the design effect can be obtained by replacing $\bar{m}$ in the design effect formula by either $\bar{m}_h$, the harmonic mean of the number of seropositive contacts per index individual, or $\bar{m} \times (1 + CV_m^2)$, where CV_m is the coefficient of variation (the standard deviation divided by the mean) of the number of seropositive contacts per index individual (5). This is particularly important when there is high overdispersion of transmission and the epidemic is in early stages, and thus, the number of seropositive contacts for each index individual will vary greatly.

Example of Sample Size Calculation. To compare the relative efficiency of a snowball sample to a random sample serosurvey, we need to compare their effective sample sizes. Assuming we perform the serology tests on a fixed sample size of individuals, *M*, then our effective sample size is Mp for a simple random sample of individuals, where p is the overall percentage of the population that is seropositive. In the snowball sampling design where the design effect is known to be *DE*, the effective sample size is Mq/DE, where q is the marginal probability of a close contact of an index individual testing positive. If $q/DE \geq p$, then the snowball sampling will be at least as efficient as the simple random sampling. If the costs of identifying index individuals and contacts leads to fewer tests available in the snowball sample, that can be incorporated as well. The snowball sampling will be at least as efficient as simple random sampling if $M_S q/DE \geq M_R p$,

where M_S is the number of tested contacts in the snowball sample and M_R is the number of tested individuals in the random sample.

As an example of this calculation, assume that 1) we identify 20 index individuals, who are independent of one another; 2) the infection has mean basic reproduction number $R_0 = 2$ and 3) the population seropositive rate is 5% (i.e., $p = 0.05$), as was estimated by seroprevalence surveys conducted in April and May 2020 in Los Angeles County, Spain, and other European countries (10–12). Now suppose that each index individual identifies 10 close contacts, each with an equal probability of being infected by the index individual. To get $R_0 = 2$, the probability of infection for each close contact is 20%, so $q \geq 0.20$. Note that the probability may be greater than 0.20 because any individual not infected by the index individual still has a chance of becoming infected later in the outbreak. Testing 200 individuals, under random sampling, will yield an expected 10 seropositive individuals. Testing 200 individuals, under snowball sampling, will yield an expected 40 seropositive individuals (not including the index cases). Assuming no overdispersion of transmission (2), the mean cluster size (excluding clusters of size 0) is 2.24 with a variance of 1.25, giving $\overline{m} \times (1 + CV_m^2) = 2.8$. So for any value of $\rho \leq 1$, $q/DE \geq p$ and the snowball sampling design is more efficient than random sampling. For $\rho = 0.05$, as used in the design of the Ebola ring vaccination trial (13), the effective sample size of the snowball sampling design is more than three times that of the random sampling design.

If the snowball design additionally tests the close contacts of all seropositive individuals found among the first ring of close contacts, then an additional 400 tests will yield 80 more seropositive individuals, provided the transmission events occurred sufficiently prior to testing for

seroconversion to occur. That is, 600 tests yield 120 seropositive individuals total, whereas randomly sampling 600 individuals to test would be expected to yield 30 seropositive individuals. Treating all individuals identified in the close contact chain of an initial index case as a cluster, this method will increase mean cluster size and thus the design effect, leading to a lower relative benefit of the snowball sampling design (although this is likely conservative as the design effect will likely decrease by using a more appropriate multi-level hierarchical model or covariance structure). But it may be a more feasible method of getting a larger snowball sample than identifying more index cases.

In the absence of serological surveys, the relative effective sample sizes of the methods would need to be estimated by the timing of the survey in the course of the outbreak. Using the generation interval, reproduction number, population size, and the number of introductions to a population, the cumulative incidence can be estimated at any point in the outbreak (14,15). This estimate can be used as p for a serological survey conducted once those infections have seroconverted, which in the case of severe acute respiratory syndrome coronavirus 2 (SARS-CoV-2) infection is estimated to occur within three weeks after symptom onset (16).

Web References

1. Lohr SL. *Sampling: Design and Analysis*. 2nd edn. Boston, MA: Brooks/Cole; 2010.

2. Dean N, Pagano M. Evaluating confidence interval methods for binomial proportions in clustered surveys. *J. Surv. Stat. Methodol.* 2015;3:484–503.

3. Commenges D, Jacqmin H. The intraclass correlation coefficient: distribution-free definition and test. *Biometrics*. 1994;50(2):517–526.

4. Eldridge SM, Ukoumunne OC, Carlin JB. The intra-cluster correlation coefficient in cluster randomized trials: a review of definitions. *Int. Stat. Rev.* 2009;77(3):378–394.

5. Rutterford C, Copas A, Eldridge S. Methods for sample size determination in cluster randomized trials. *Int. J. Epidemiol.* 2015;44(3):1051–1067.

6. Agresti A. *Categorical Data Analysis*. 2nd edn. Hoboken, NJ: John Wiley & Sons, 2003.

7. Stiratelli R, Laird N, Ware JH. Random-effects models for serial observations with binary response. *Biometrics*. 1984;40(4):961–971.

8. Hedeker D. A mixed-effects multinomial logistic regression model. *Stat. Med.* 2003;22(9):1433–1466.

9. Zeger SL, Liang KY. Longitudinal data analysis for discrete and continuous outcomes. *Biometrics*. 1986;42(1):121–130.

10. Sood N, Simon P, Ebner P. Seroprevalence of SARS-CoV-2-specific antibodies among adults in Los Angeles County, California, on April 10–11, 2020. *JAMA*. 2020;323(23):2425–2427.

11. Pollán M, Pérez-Gómez B, Pastor-Barriuso R, et al. Prevalence of SARS-CoV-2 in Spain (ENE-COVID): a nationwide, population-based seroepidemiological study. *Lancet*. 2020;396(10250):535–544.

12. Okell LC, Verity R, Watson OJ, et al. Have deaths from COVID-19 in Europe plateaued due to herd immunity? *Lancet.* 2020;395:e110–e111.

13. Ebola ça suffit ring vaccination trial consortium. The ring vaccination trial: a novel cluster randomised trial design to evaluate vaccine efficacy and effectiveness during outbreaks, with special reference to Ebola. *BMJ*. 2015;351:h3740.

14. Wallinga J, Lipsitch M. How generation intervals shape the relationship between growth rates and reproductive numbers. *Proc. R. Soc. B.* 2007;274:599–604.

15. Anderson RM, May RM. *Infectious Diseases of Humans: Dynamics and Control*. Oxford, UK: Oxford University Press; 1991.

16. Long Q-X, Liu B-Z, Deng H-J, et al. Antibody responses to SARS-CoV-2 in patients with COVID-19. *Nat. Med.* 2020;26:845–848.

Web Table 1. Summary of parameters used in simulations

Parameter	Values Used
Initial Population	9,994 Susceptible<br>6 Infectious<br>0 Recovered
Basic Reproduction Number (R_0)	2.5
Infectious Period	Days 2–9 after infection, equal probability of transmission per day
Average Number of Contacts During Infectious Period	20
Overdispersion of Transmission, *k*	0.1, 0.5, 1.0
Proportion at High Risk of Symptom of Interest	20%
Probability of Symptom of Interest	5% Overall:<br>8% if at High Risk<br>4.25% if not at High Risk
Intracluster Correlation Coefficient (ICC) of Symptom of Interest between Infector and Infectee	0.00, 0.05 (Figures 1,2; Web Figures 1,2,4,5)<br>0.10 (Figure 3; Web Figures 3,6)
Sampling Times	25, 35, and 45 Days after Infection
Regular Sampling	600 Individuals Chosen Uniformly
Snowball Sampling	30 Index Individuals Chosen Uniformly among Individuals who Had Been Infected
Snowball, Contact Error	Per Index Individual:<br>2 True Contacts Missed<br>2 False Contacts Identified

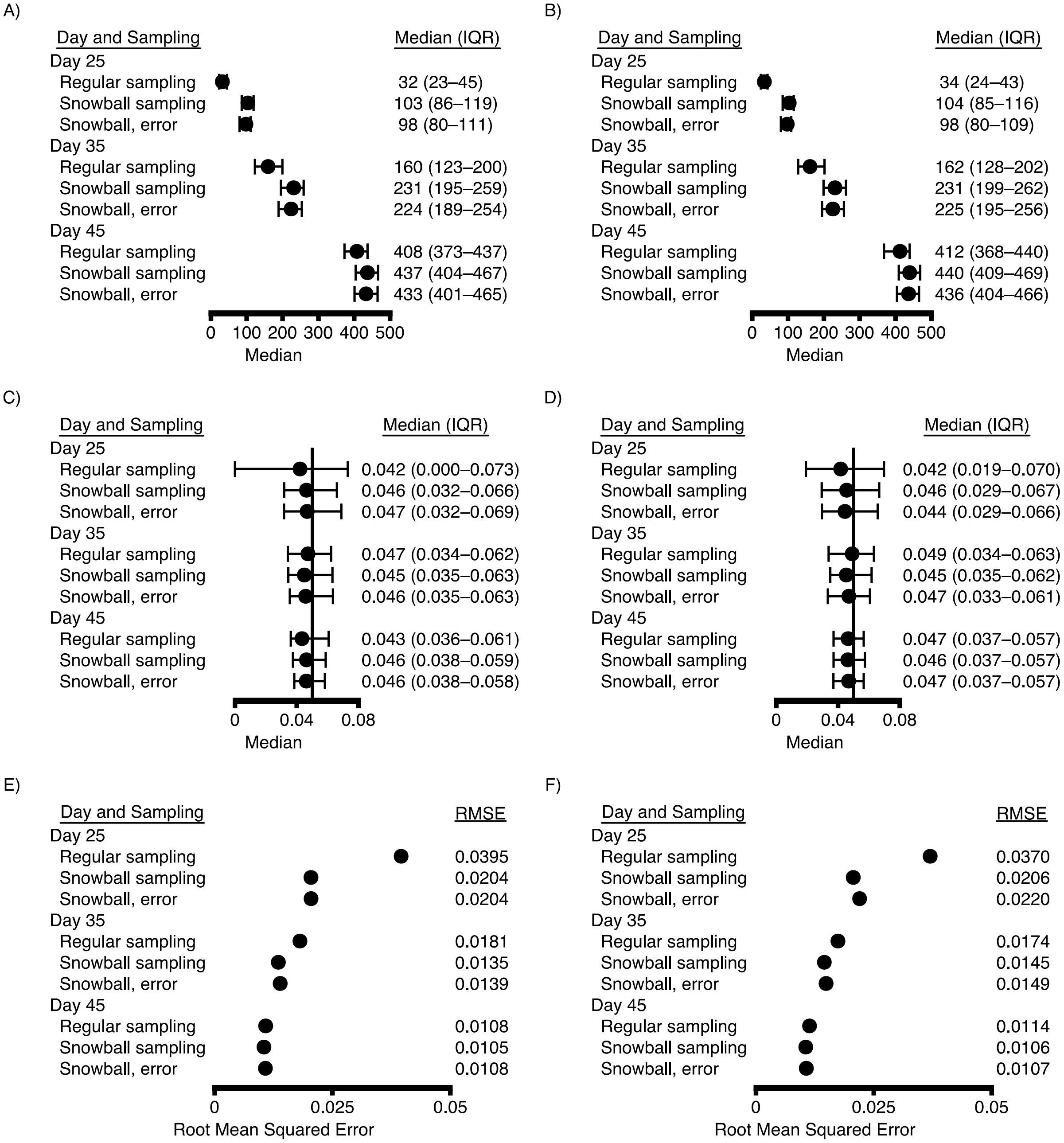

Web Figure 1. Simulation results for estimated symptom rates. Median and interquartile range (IQR) of the number of infections per sample (A,B), median and IQR of the estimated symptom rate (C,D), and root mean squared error (RMSE) of the estimated symptom rate (E,F) are compared by sampling time (day 25, 35, and 45), intracluster correlation (ICC) of infector and infectee symptom status (ICC = 0 in A,C,E and ICC = 0.05 in B,D,F), and sampling method, with dispersion parameter k = 0.5. The underlying probability of being symptomatic given infection is 5% (vertical line in C,D). All symptom rates are estimated using the logit transformation; estimates for the two snowball samples are adjusted for clustering by the index individual, with contacts named by two or more index individuals removed. Results are from 250 simulations per parameter combination.

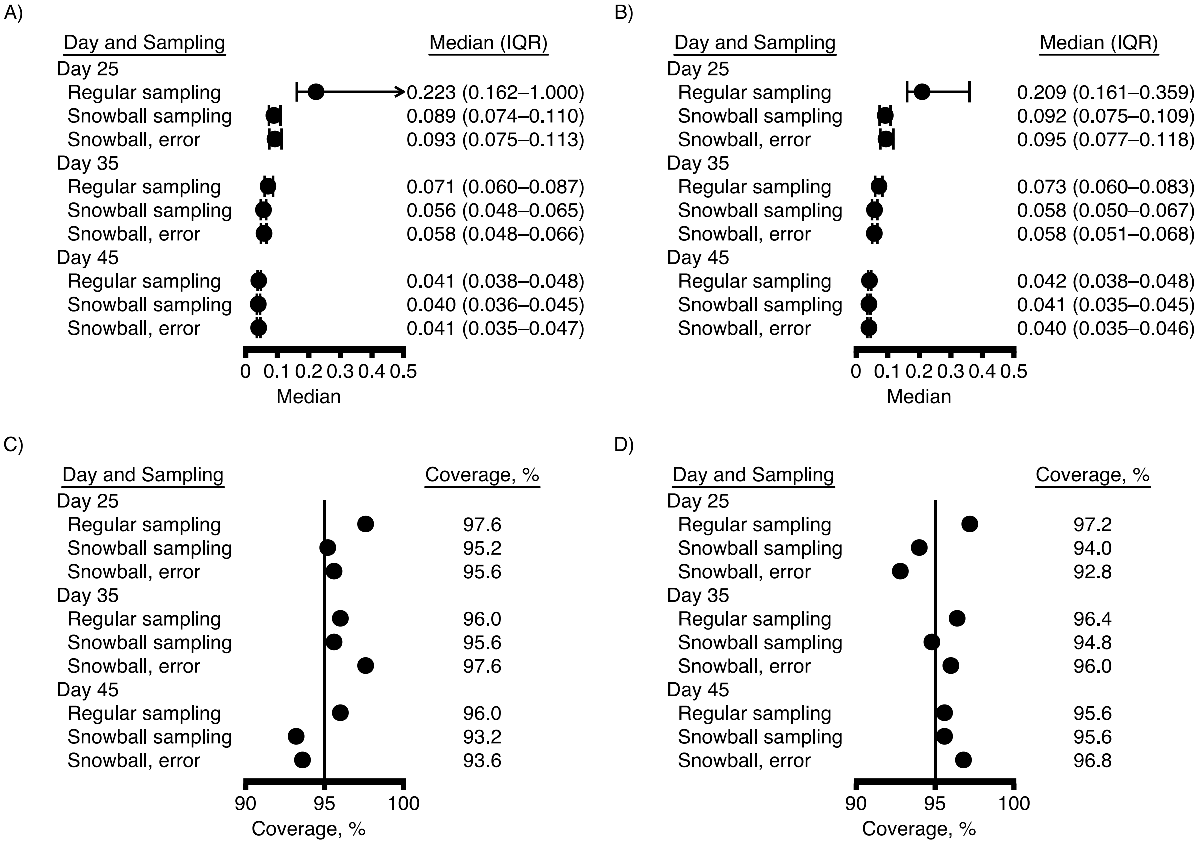

Web Figure 2. Simulation results for inference on estimated symptom rates. Median and interquartile range (IQR) of the 95% confidence interval (CI) width (A,B) and empirical coverage (C,D) of the 95% CIs for the symptom rate among infected individuals are compared by sampling time (day 25, 35, and 45), intracluster correlation (ICC) of infector and infectee symptom status (ICC = 0 in A,C and ICC = 0.05 in B,D), and sampling method, with dispersion parameter k = 0.5. The nominal CI coverage is 95% (vertical line in C,D). Results are from 250 simulations per parameter combination.

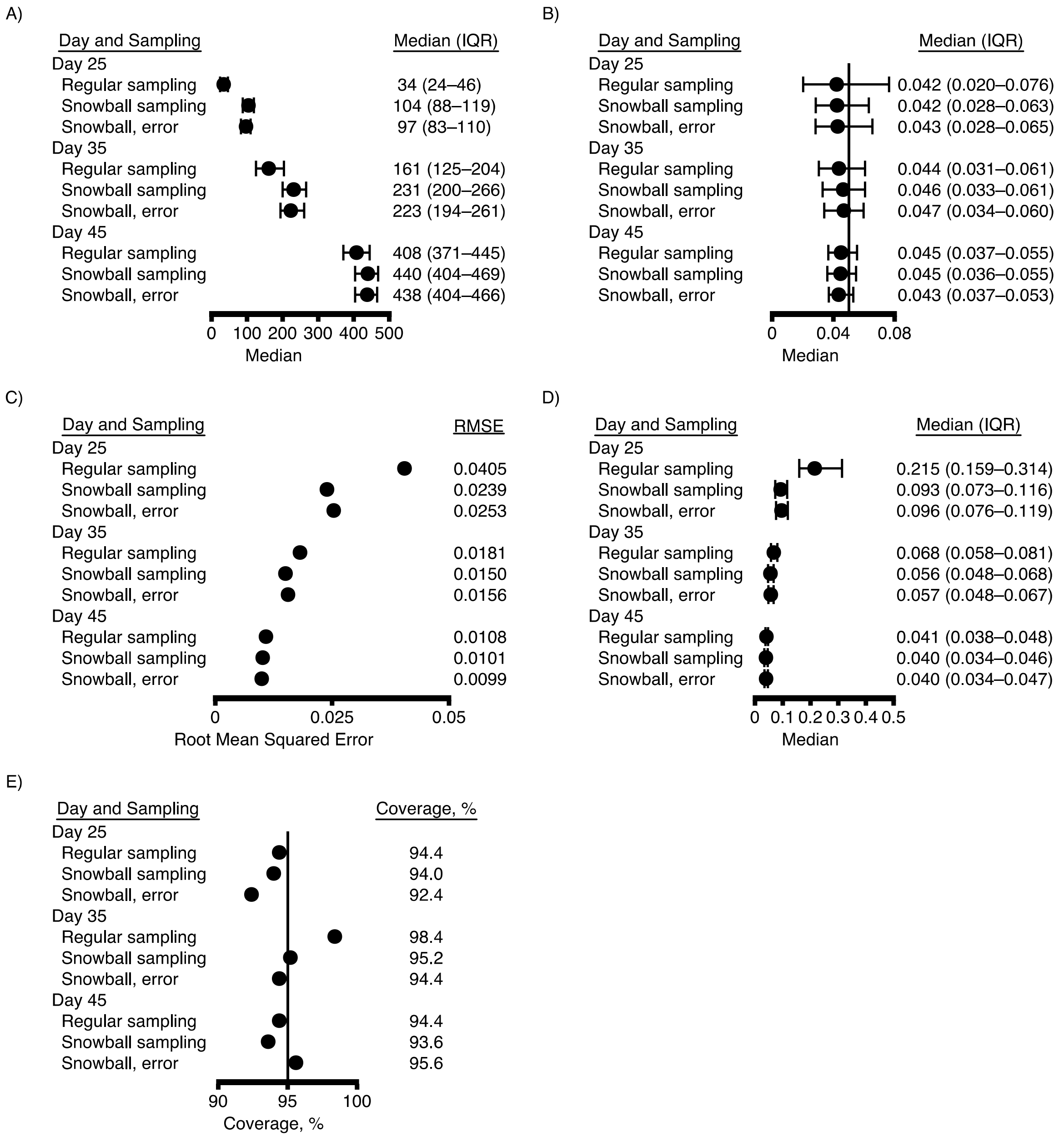

Web Figure 3. Simulation results for estimation and inference of symptom rates for intracluster correlation (ICC) of 0.10. Median and interquartile range (IQR) of the number of infections per sample (A), median and IQR of the estimated symptom rate (B), root mean squared error (RMSE) of the estimated symptom rate (C), median and IQR of the 95% confidence interval (CI) width (D) and empirical coverage (E) of the 95% CIs for the symptom rate are compared by sampling time (day 25, 35, and 45) and sampling method, with dispersion parameter k = 0.5 and with ICC = 0.10. The underlying probability of being symptomatic given infection is 5% (vertical line in B) and the nominal CI width is 95% (vertical line in E). All symptom rates are estimated using the logit transformation; estimates for the two snowball samples are adjusted for clustering by the index individual, with contacts named by two or more index individuals removed. Results are from 250 simulations per parameter combination.

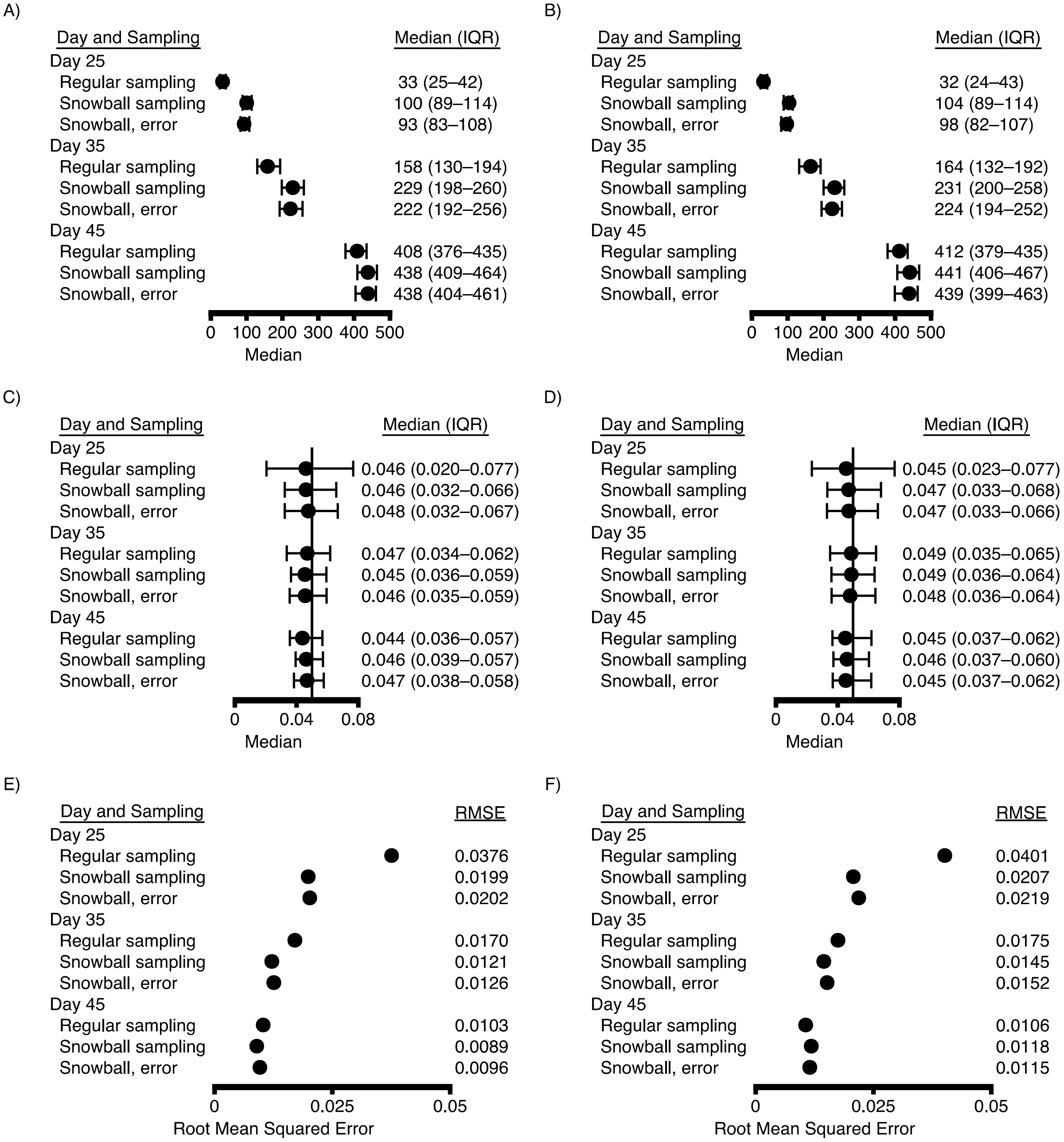

Web Figure 4. Simulation results for estimated symptom rates. Median and interquartile range (IQR) of the number of infections per sample (A,B), median and IQR of the estimated symptom rate (C,D), and root mean squared error (RMSE) of the estimated symptom rate (E,F) are compared by sampling time (day 25, 35, and 45), intracluster correlation (ICC) of infector and infectee symptom status (ICC = 0 in A,C,E and ICC = 0.05 in B,D,F), and sampling method, with dispersion parameter k = 1.0. The underlying probability of being symptomatic given infection is 5% (vertical line in C,D). All symptom rates are estimated using the logit transformation; estimates for the two snowball samples are adjusted for clustering by the index individual, with contacts named by two or more index individuals removed. Results are from 250 simulations per parameter combination.

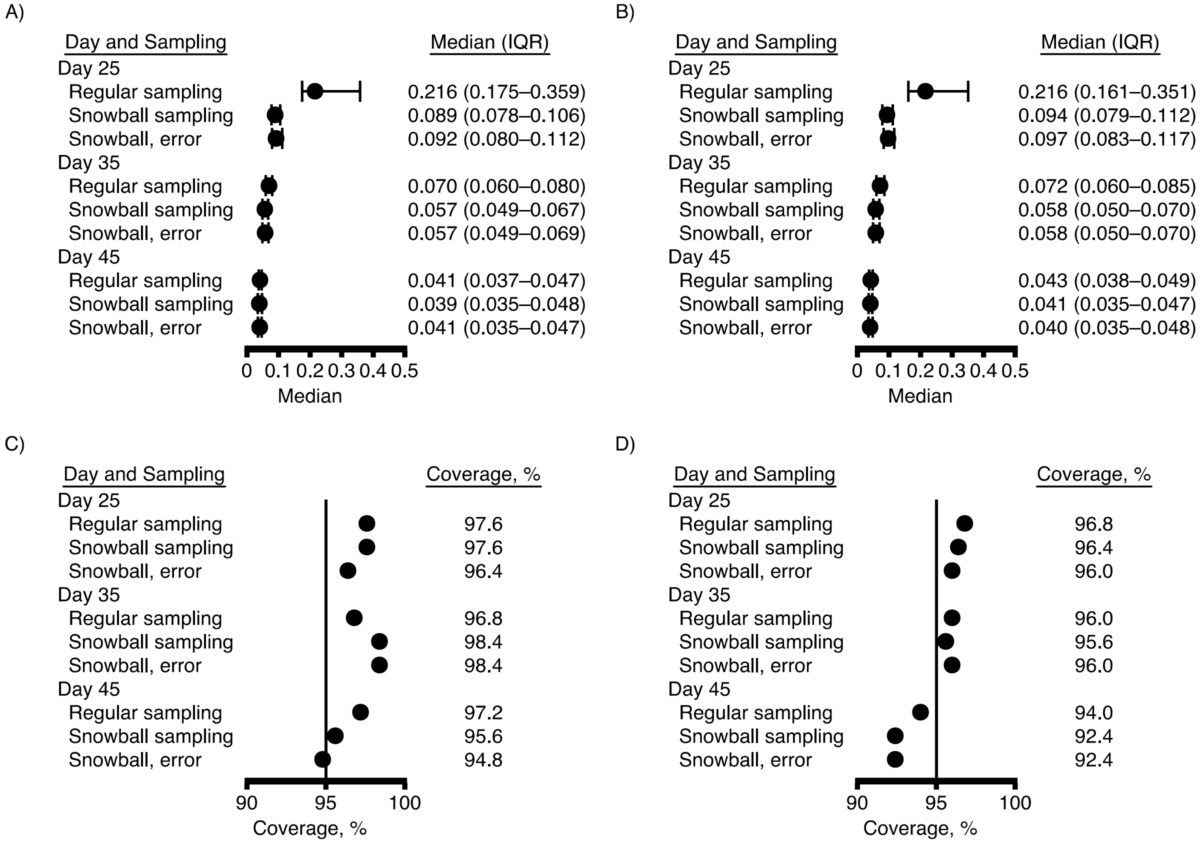

Web Figure 5. Simulation results for inference on estimated symptom rates. Median and interquartile range (IQR) of the 95% confidence interval (CI) width (A,B) and empirical coverage (C,D) of the 95% CIs for the symptom rate among infected individuals are compared by sampling time (day 25, 35, and 45), intracluster correlation (ICC) of infector and infectee symptom status (ICC = 0 in A,C and ICC = 0.05 in B,D), and sampling method, with dispersion parameter k = 1.0. The nominal CI coverage is 95% (vertical line in C,D). Results are from 250 simulations per parameter combination.

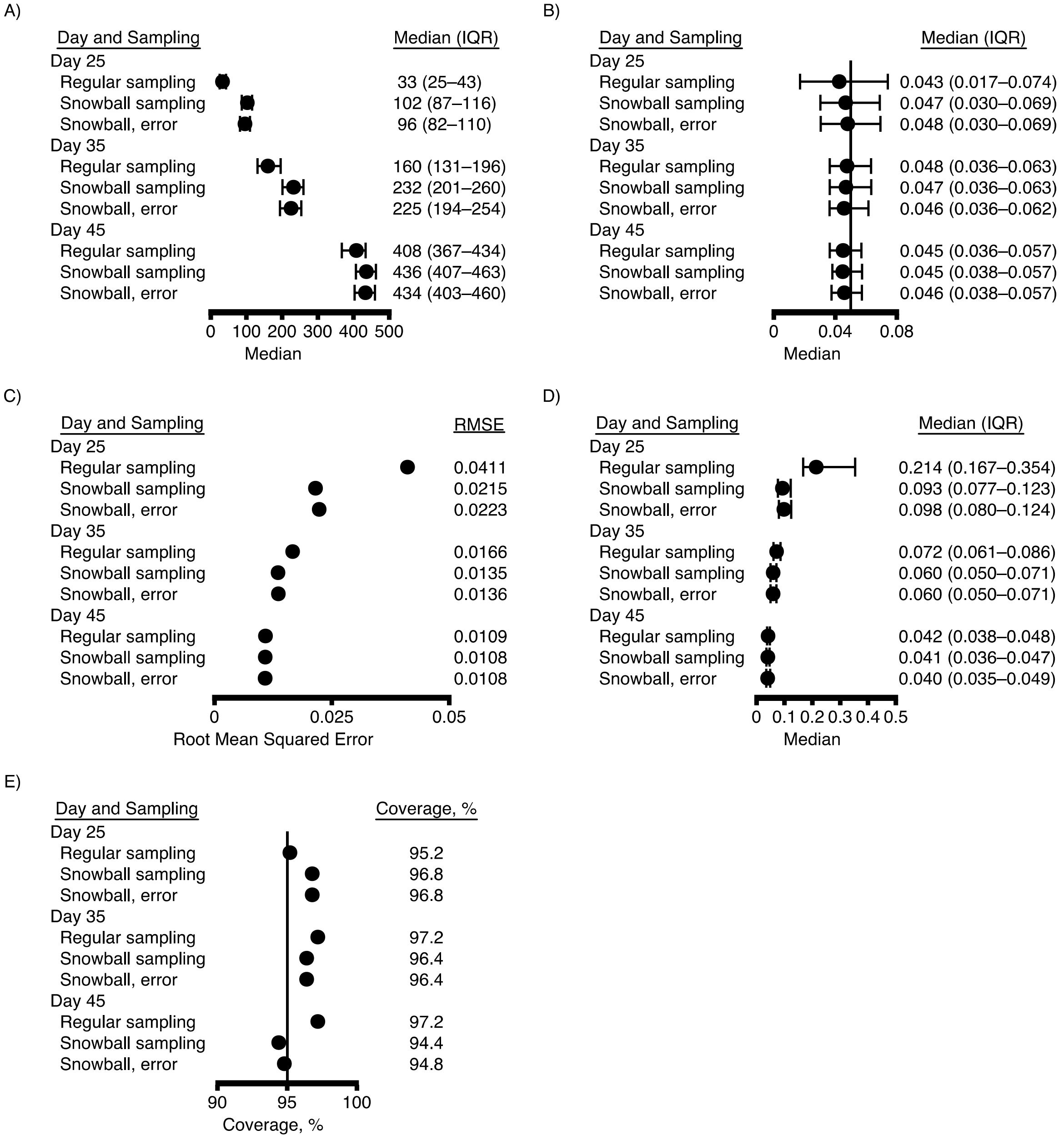

Web Figure 6. Simulation results for estimation and inference of symptom rates for intracluster correlation (ICC) of 0.10. Median and interquartile range (IQR) of the number of infections per sample (A), median and IQR of the estimated symptom rate (B), root mean squared error (RMSE) of the estimated symptom rate (C), median and IQR of the 95% confidence interval (CI) width (D) and empirical coverage (E) of the 95% CIs for the symptom rate are compared by sampling time (day 25, 35, and 45) and sampling method, with dispersion parameter k = 1.0 and with ICC = 0.10. The underlying probability of being symptomatic given infection is 5% (vertical line in B) and the nominal CI width is 95% (vertical line in E). All symptom rates are estimated using the logit transformation; estimates for the two snowball samples are adjusted for clustering by the index individual, with contacts named by two or more index individuals removed. Results are from 250 simulations per parameter combination.
